# Clinical Associations of Biallelic and Monoallelic* TNFRSF13B* Variants in Italian Primary Antibody Deficiency Syndromes

**DOI:** 10.1155/2016/8390356

**Published:** 2016-03-30

**Authors:** Federica Pulvirenti, Roberta Zuntini, Cinzia Milito, Fernando Specchia, Giuseppe Spadaro, Maria Giovanna Danieli, Andrea Pession, Isabella Quinti, Simona Ferrari

**Affiliations:** ^1^Department of Molecular Medicine, Sapienza Medical University, Viale Università 37, 00185 Rome, Italy; ^2^Department of Medical Genetics, Policlinico S. Orsola-Malpighi, Medical University of Bologna, Via Massarenti 9, 40138 Bologna, Italy; ^3^Department of Pediatrics, Policlinico S. Orsola-Malpighi, Medical University of Bologna University of Bologna, Via Pietro Albertoni 15, 10138 Bologna, Italy; ^4^Department of Clinical Medicine and Surgery, Medical University of Naples Federico II, Corso Umberto I 40, 80138 Naples, Italy; ^5^Department of Medical and Molecular Sciences, Torrette Hospital, Medical University of Ancona, Via Conca 71, 60126 Torrette, Italy

## Abstract

We assessed the prevalence of* TNFRSF13B *mutations and the clinical correlates in an Italian cohort of 189 CVID, 67 IgAD patients, and 330 healthy controls to substantiate the role of TACI genetic testing in diagnostic workup. We found that 11% of CVID and 13% of IgAD carried at least one mutated* TNFRSF13B *allele. Seven per cent of CVID had monoallelic-mutations and 4% had biallelic-mutations. The frequency of C104R monoallelic-mutations was not higher than that found in healthy controls. Biallelic-mutations were exclusively found in CVID. CVID patients carrying monoallelic-mutations had an increased prevalence of lymphadenopathy, granulomata, and autoimmune cytopenias. CVID carrying biallelic-mutations had a low prevalence of autoimmunity in comparison with TACI wild-type CVID. Moreover, biallelic-mutated CVID had higher frequency of switched memory B-cells and higher IgM and IgA antibodies to polysaccharide antigens than TACI wild-type and monoallelic-mutated CVID. TACI-mutated IgAD patients had only monoallelic-mutations and did not display clinical difference from IgAD wild-type patients. In conclusion,* TNFRSF13B *genetic screening of antibody deficiencies may allow the identification of mutational patterns. However, as with counseling for risk assessment, geneticists should be aware that the interpretation of genetic testing for TACI mutations is difficult and the potential impact on clinical management is still limited.

## 1. Introduction

Defects in antibody production predisposing to various types of infections are the hallmark of primary antibody deficiencies (PADs) [1, 2]. Genetic defects underlying PADs remain unknown in the majority of cases. Recently, the European ESID Registry has defined Working Definitions for Clinical Diagnosis of PAD entities for patients without genetic diagnosis including Common Variable Immunodeficiency Disorders (CVID), the most common symptomatic antibody deficiency, and Selective IgA Deficiency (IgAD) (http://esid.org/). In the absence of genetic mutation(s) these criteria are based on clinical and immunological abnormalities, allowing a “possible” and not a “definitive” diagnosis. The clinical spectrum of PADs varies between different entities and even within the same entity, ranging from severe disease with a reduced life expectancy, to very mild or even asymptomatic forms. For CVID the clinical phenotype ranges from a poorly symptomatic one to severe phenotypes characterized by high susceptibility to infections, autoimmunity, granulomatous inflammation, lymphoproliferative disorders, and malignancies [3–8].

Immunologic abnormalities in patients with CVID include defects of B-cell survival, decreased frequency of circulating CD27^+^ memory B-cells, failure of isotype switching to IgA and IgG, defective B-cell activation, and inability to mount responses to polysaccharide antigens [9].

The search for causative or susceptibility gene(s) is progressing and rare autosomal recessive mutations in a number of single genes have recently been reported in CVID [8]. In particular, mutations in the* TNFRSF13B* gene encoding TACI, a tumor necrosis factor receptor superfamily member expressed on B-cells, have been reported in 7–10% of CVID patients [10–12]. Similarly, TACI mutations have also been observed in IgAD [10, 11, 13, 14]. TACI can bind two ligands, a proliferation-inducing ligand (APRIL) and B-cell-activating factor (BAFF) [15], and is critical for B-cell homeostasis and function. TACI is essential for the establishment of central B-cell tolerance, given that all subjects carrying C104R and A181E missense* TNFRSF13B* mutations have an inability to remove developing autoreactive B-cells in bone marrow [16]. Intracellular TACI domains interact with several signaling molecules, including myeloid differentiation factor 88 (MyD88) gene, as well as activated endosomal toll-like receptor (TLR) 7 and TLR9 [17, 18].

Several papers have analyzed the pathogenic role of* TNFRSF13B* mutations in B-cell function, development, and tolerance.* TNFRSF13B* haploinsufficiency or null alleles result in decreased TACI expression on memory B-cells and impaired antibody secretion, suggesting that during later stages of B-cell development, TACI supports class-switch recombination, plasma cell differentiation, and antibody secretion [19, 20].

The role of TACI in T cell-independent antibody response is controversial [21–24]. For the majority of the authors the knockout of* TNFRSF13B* gene in mice results in an impaired T cell-independent type II (TI-2) response and virtually abolishes APRIL-induced switching to IgA, IgE, and IgG1 [21, 22]. In addition, TACI^−/−^ mice spontaneously develop lymphoproliferation and a lethal autoimmune syndrome [25].

Many cohort studies have screened PAD patients for TACI mutations [12, 13, 26–28], mainly in exons 3 and 4 because the vast majority of all detected mutations, including a C104R mutation that alters ligand binding and the A181E mutation that affects transmembrane function [29, 30], occur in these exons. Compound heterozygotes and homozygotes have been identified, but in the majority of cases* TNFRSF13B* mutations are present as simple heterozygous variants. There is a general agreement that, in CVID, monoallelic mutations are associated with autoimmunity and lymphoproliferation phenotype [12, 16], while few studies have addressed the issue of TACI mutations and their clinical significance in IgAD [13, 14, 31]. The clinical and immunological associations of biallelic TACI mutations are less clear [13]. At present, it is doubtful whether detection of TACI mutations could be helpful for early diagnosis and prognosis in affected patients. In our study, we examined the prevalence of TACI mutations and their clinical correlates in a population of Italian CVID and IgAD patients, in order to evaluate whether screening for TACI mutations should be recommended as part of the genetic diagnostic workup and genetic counseling.

## 2. Methods

### 2.1. Patients

We enrolled 256 adult Caucasian patients with PADs diagnosed according to ESID criteria [1], 189 of whom were affected by CVID and 67 by IgAD. Patients were attending the clinics for Primary Immunodeficiencies from four Italian cities: Rome, Naples, Ancona, and Bologna. We also included in the study 330 Caucasian anonymous healthy adult donors >50 years old, recruited from Italian Blood Donor Centers. Relevant clinical and immunological data were collected from medical files, including serum immunoglobulin (Ig) levels at diagnosis, clinical history of recurrent infections, chronic diarrhea, bronchiectasis, autoimmune diseases (autoimmune hemolytic anemia (AHA), idiopathic thrombocytopenic purpura (ITP), vitiligo, arthritis, coeliac disease (CD), insulin dependent diabetes mellitus (IDDM), atrophic gastritis, inflammatory bowel diseases (IBD)), lymphoproliferative disorders (splenomegaly, lymph nodes enlargement, and granulomatous disease), and cancers. For CVID patients only, laboratory assessment of the frequency of T cell and B-cell subsets and the response to pneumococcal polysaccharide antigens were collected. The institutional review board approved the study and a signed informed consent was obtained from all participants.

### 2.2. Sequence Analysis of* TNFRSF13B*

PCR amplification and sequencing of* TNFRSF13B* exons and splicing junctions were performed with primers and conditions as described in Salzer et al. [10]. Sequence analysis was performed using Sequencer version 5.0 (Gene Codes Corporation, Ann Arbor, MI, USA).

To estimate the pathogenic effect of the described* TNFRSF13B* mutations on protein structure and function, we employed web-based* in silico* software tools. The impact of mutations on protein structure was assessed with PolyPhen2 (http://genetics.bwh.harvard.edu/pph2/) and on splicing with Human Splicing Finder 3.0 (http://www.umd.be/HSF3/HSF.html).

### 2.3. Flow Cytometry Analysis

Peripheral blood mononuclear cells were obtained by density-gradient centrifugation. Immunophenotyping was performed with a combination of 4 fluorochrome-labeled monoclonal antibodies (BD Biosciences). The following B-cell populations were analyzed: classical naïve (CD19^+^CD27^−^CD21^+^CD38^+^), switched memory (CD19^+^CD27^+^CD21^+^IgM^−^), IgM memory (CD19^+^CD27^+^IgM^+^IgD^+^), and transitional (CD19^+^IgM^++^CD38^++^) and CD21 low (CD19^+^CD21^−/low^CD38^−^). The following T cell subsets were analyzed: CD4 (CD3^+^CD4^+^), CD8 (CD3^+^CD8^+^), CD4 memory (CD4^+^CD45RO^+^), CD4 naïve (CD4^+^CD45RA^+^), and CD4 Treg (CD4^+^CD25^high^CD127^−^). Dead cells were excluded from analysis by side/forward scatter gating. FACS analyses were performed on a FACSCalibur instrument (BD Biosciences) using Cell Quest (BD) and FlowJo (Tree Star) software.

### 2.4. 23 Serotype-Specific Anti-Pneumococcal Polysaccharide IgM and IgA Antibodies

IgM and IgA antibodies to 23 PS serotypes were quantified using a new ELISA test PS23 IgA and PS23 IgM ELISA, modified from the commercially available PS23 Pneumococcal Capsular Polysaccharide IgG VaccZyme*™* ELISA, as described in our previous papers [32, 33].

### 2.5. Statistical Analysis

Statistical analysis was performed with dedicated software (StatView, GraphPad). Descriptive data are presented as mean and standard deviation (SD). The statistical significance of differences in the frequencies of mutations and polymorphisms between groups was evaluated using two-tailed Fisher's exact test. Comparison of clinical features between groups was performed by the Mann-Whitney test. Comparison of categorical and noncontinuous variables between groups was performed by Fisher's exact test. Comparison of polysaccharide antibodies before and after immunization was performed by the Wilcoxon test. A *p* value of <0.05 was taken as the threshold of statistical significance.

## 3. Results

### 3.1. Clinical Characteristics of the PAD Population

*(a) CVID Patients*. One hundred eighty-nine (92 males and 97 females) CVID patients were enrolled in the study. The mean age at the time of the study was 51 ± 15.6 years. Immunoglobulin levels at diagnosis were IgG 272 ± 153 mg/dL; IgA 25 ± 31 mg/dL; and IgM 73 ± 170 mg/dL. At the time of the study all patients were on Ig replacement therapy and their serum IgG trough level was 682 ± 195 mg/dL. One hundred five (56%) patients had previously almost one episode of pneumonia, 85 (45%) had bronchiectasis, and 47 (25%) had chronic diarrhea.

Fifty patients (27%) had at least one autoimmune manifestation: 7% had AHA, 12% had ITP, and 18% had other autoimmune manifestations (including vitiligo, arthritis, CD, IDDM, atrophic gastritis, and IBD). Signs of lymphoproliferative disorders were found in 114 patients (60%): splenomegaly was found in 59% of patients, lymph nodes enlargement in 28%, and granulomatous disease in 15%. Eighty-five patients had bronchiectasis (45%) and 47 patients (25%) had chronic diarrhea. Thirty-three patients (17.5%) had a diagnosis of cancer and three patients had more than one cancer: 12 patients had B-cell non-Hodgkin lymphoma; two patients had splenic lymphoma; 6 patients had gastric adenocarcinoma, two patients had colorectal adenocarcinoma, and 15 patients had other cancers. Among the 189 patients, 180 cases were sporadic and 9 were familiar from 5 families.

*(b) IgAD Patients*. Sixty-seven IgAD patients (44 males and 23 females) with mild symptomatic upper respiratory tract infections were enrolled. The mean age at the study time was 11.3 ± 10.6 years. Immunoglobulins levels at diagnosis were IgG 1372.9 ± 333.1 mg/dL; IgA 6.5 ± 8.2 mg/dL; IgM 119.0 ± 41.7 mg/dL. Sixteen patients (24%) had at least one autoimmune manifestation, including CD (7 patients), tiroiditis (4 patients), juvenile idiopathic arthritis (1 patients), IDDM (1 patient), and vasculitis (1 patient). None had autoimmune cytopenias or signs of neither lymphoproliferative disorders nor cancer.

Among the 67 index patients, 55 cases were sporadic and 12 were familial from 5 families.

### 3.2. *TNFRSF13B* Genetic Analysis of the PAD Population

**Figure 1:**
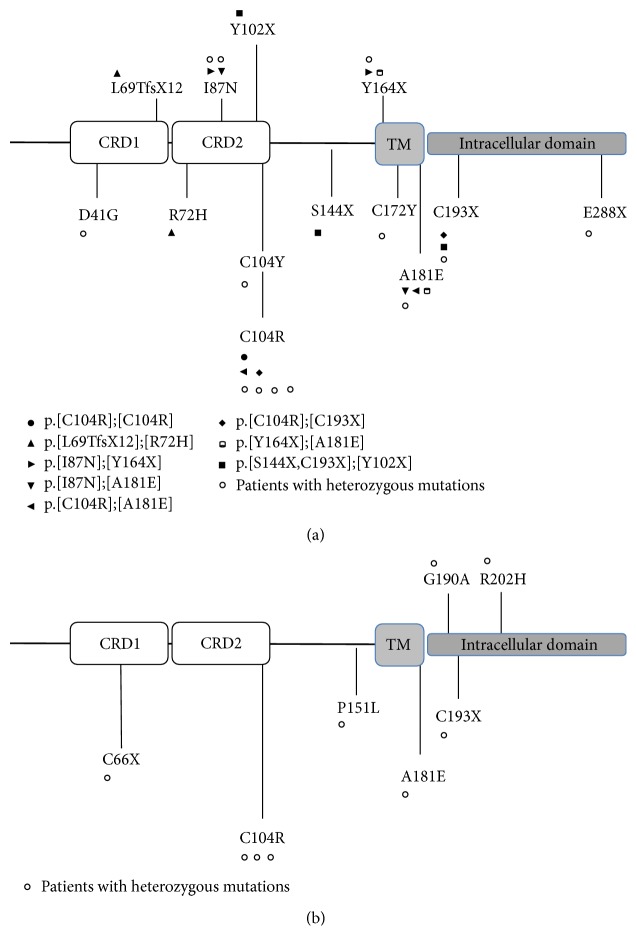
Distribution of* TNFRSF13B* mutations across the TACI protein in 21 of 189 patients with CVID (a) and in 9 of 67 patients with IgAD (b). Each symbol represents one patient. ● indicates patients with homozygous* TNFRSF13B* mutations; ∘ represents patients with heterozygous* TNFRSF13B* mutations. Compound heterozygous mutated TACI-deficient patients are labeled as indicated in the figure. CRD1 and CRD2 indicate cysteine-rich domains (residues 32–68 and 69–107); TM, transmembrane domain (residues 160–182).

We found that 30/256 PAD patients (11.8%) carried at least one* TNFRSF13B* variant (Figures 1(a) and 1(b) and Table 1).

Twenty-one CVID patients (11.1%) carried at least one* TNFRSF13B* variant. Monoallelic mutations were found in 13 patients (6.9%): 4 patients (2.3%) carried the heterozygous C104R mutation and 9 patients (5.1%) had non-C104R monoallelic mutations. Eight patients (4.5%) carried biallelic mutations including one patient carrying the C104R homozygous variant. Seven patients carried compound heterozygous mutations: [L69TfsX12];[R72H], [I87N];[Y164X], [I87N];[A181E], [C104R];[C193X], [C104R];[A181E], [Y164X];[A181E], and [S144X,C193X];[Y102X] (Table 1(a) and Figure 1).

Nine IgAD patients (13.4%) carried a single* TNFRSF13B* variant: 3 patients (4.7%) were sibs and had the heterozygous C104R mutation and 6 patients (9.4%) carried non-C104R monoallelic mutations. None of the IgAD patients had biallelic mutations (Table 1(b)). A total of 330 controls were analyzed for sequence variants in* TNFRSF13B*. Two of them (0.9%) were heterozygous for the C104R variant and one (0.4%) was heterozygous for the I87N allele. No other monoallelic or biallelic missense mutations were found in healthy controls.

The frequency of* TNFRSF13B* gene mutations was significantly higher in CVID compared to healthy controls (*p* < 0.0001), with the exception of monoallelic C104R mutations (*p* = ns) (Table 1(a)). The frequency of TACI mutations was significantly higher in IgAD compared to healthy controls, either including or not the three sibs carrying the C104R mutation (*p* = 0.0001 and *p* = 0.0005, resp.) (Table 1(b)).

Among PAD patients carrying variants, 25 were unrelated and 5 belonged to two different families: three sibs with IgAD were heterozygous for C104R and two sibs with CVID were heterozygous for I87N. In both cases the mutation was inherited from one unaffected parent.

Seventeen different genetic alterations were observed, one of which has not been previously described: a novel nonsynonymous G190A variant found in an IgAD patient. The polymorphisms V220A (rs56063729, MAF 0.016) and P251L (rs34562254, MAF 0.139) were observed in the cohort of antibody-deficient patients at frequencies that were not significantly different from those observed in healthy control populations.

All the nonsynonymous variants, with the exception of G190A, were present in dbSNP with a MAF ranging from <0.0001 to 0.005 (Table 2). The possible impact of each amino acid substitution on structure and function of the protein was predicted using PolyPhen2. All the missense variants were predicted to be damaging with a score ranging from 0.474 to 1.000, with the exception of R72H, predicted to be benign with a score of 0.003. The Human Splicing Finder 3.0 tool predicted that the nucleotide substitution leading to Arg > His amino acid change at the position 72 of the protein (c.215 G > A) broke an Exonic Splicing Enhancer (ESE) binding site for SF2/ASF protein located at the position +11 to +17 from the exon 3 junction, potentially altering the splicing of the corresponding exon. Hence, we decided to include the compound heterozygous patient, carrying both R72H and L69TfsX12 variants (I.74 in Table 3), in the group of patients with biallelic genetic alterations. All missense mutations are classified by ClinVar as Variants of Uncertain Significance (VUS), with the exception of C104R and A181E whose pathogenicity has already been proven.

### 3.3. Clinical Phenotypes Observed in TACI-Mutated CVID

Clinical details of 21 TACI-mutated CVID patients are reported in Table 3. A summary of the frequency of clinical manifestations is reported in Table 4.

**Figure 2:**
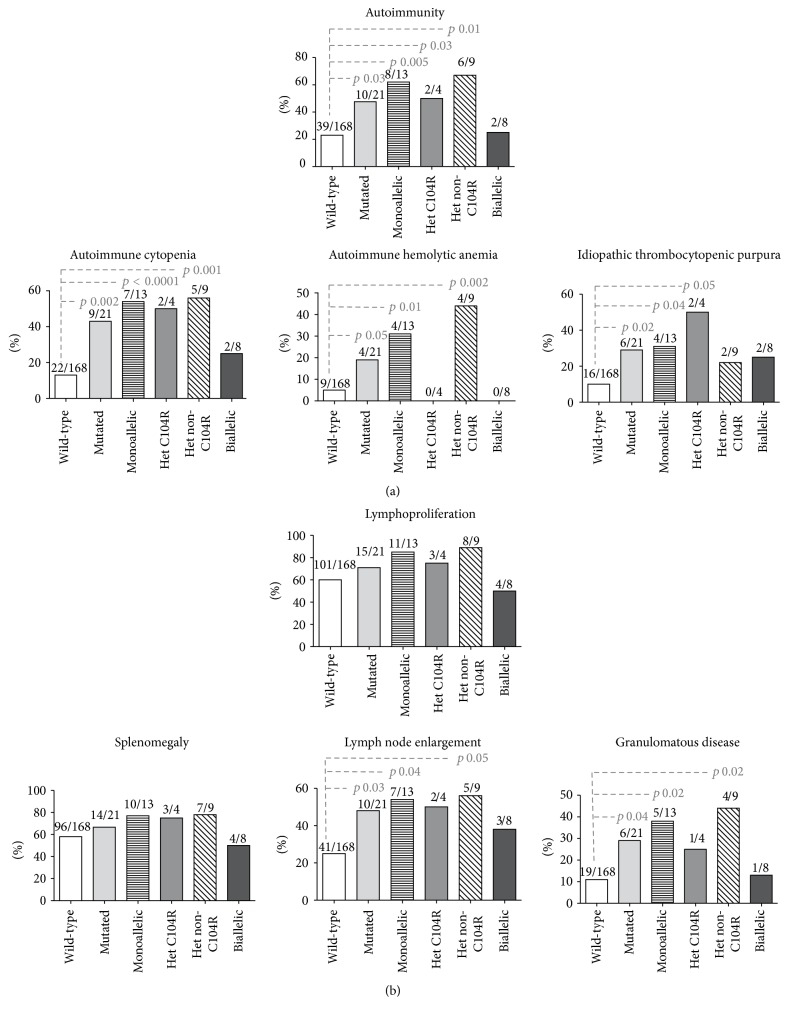
Autoimmune (a) and lymphoproliferative (b) complications in patients with TACI wild-type or mutated sequence. TACI-deficient individuals were grouped as carrying monoallelic TACI mutations (heterozygous C104R and heterozygous non-C104R) and biallelic TACI mutations.

*(a) Monoallelic TNFRSF13B Mutations*. As already demonstrated [12, 34] CVID patients carrying* TNFRSF13B* heterozygous mutations had a higher prevalence of autoimmunity compared with patients with wild-type* TNFRSF13B* (61.5% versus 23%, *p* = 0.005) (Figure 2(a)). Autoimmune cytopenia was the most common autoimmune phenomenon in patients with heterozygous* TNFRSF13B* mutations. CVID patients with monoallelic TACI mutations had a higher frequency of autoimmune cytopenias than TACI wild-type patients (54% versus 13%, *p* < 0.0001); in particular, patients with* TNFRSF13B* C104R mutations had a higher prevalence of ITP (50% versus 10%, *p* = 0.05), whereas patients with non-C104R* TNFRSF13B* mutations had a higher frequency of AHA (44% versus 5%, *p* = 0.002). The overall prevalence of lymphoproliferative manifestations was similar among CVID groups (Figure 2(b)). However, lymph node enlargement and granulomatous diseases were more frequently observed in monoallelic TACI-mutated CVID compared to wild-type CVID (54% versus 25%, *p* = 0.04 and 38% versus 11%, *p* = 0.02, resp.). No difference was observed between TACI wild-type CVID and CVID carrying monoallelic TACI mutations on the frequencies of infective manifestations, including pneumonia, chronic diarrhea, and the presence of bronchiectasis.

*(b) Biallelic TNFRSF13B Mutations*. No clinical differences were observed between CVID carrying* TNFRSF13B* biallelic mutations and wild-type. In particular, the prevalence of autoimmunity was similar between the groups (25% versus 23%, *p* value being nonsignificant) (Figure 2(a)). No difference was observed between patients carrying biallelic or monoallelic TACI mutations on the presence of bronchiectasis and on frequencies of infective manifestations (episode of pneumonia/chronic diarrhea). A malignant lymphoproliferative disease was recorded in a single TACI-mutated CVID patient suffering from indolent B-cell non-Hodgkin lymphoma and carrying biallelic mutations [L69TfsX12];[R72H].

### 3.4. Immunological Phenotype and Immunoglobulin Levels in CVID TACI-Mutated Patients

*(a) Monoallelic TNFRSF13B Mutations*. A severe B-cell lymphopenia (<1%) was identified only in one TACI-mutated patient (C193X). The frequency of IgM^−^IgD^−^CD27^+^ switched memory B-cells was similar in CVID carrying monoallelic TACI mutations and in TACI wild-type (5.6%  ±  4.4 versus 3.4%  ± 5), while the frequency of IgD^+^IgM^+^CD27^−^ naïve B-cells was lower (53.8%  ± 33.6 versus 76.2%  ± 19.2, *p* = 0.01). Among TACI-mutated patients, the group carrying a non-C104R heterozygous mutation had the lowest B naïve frequency compared to wild-type subjects (37.5%  ± 53). No differences were found in the frequency of CD21low cells.

**Figure 3:**
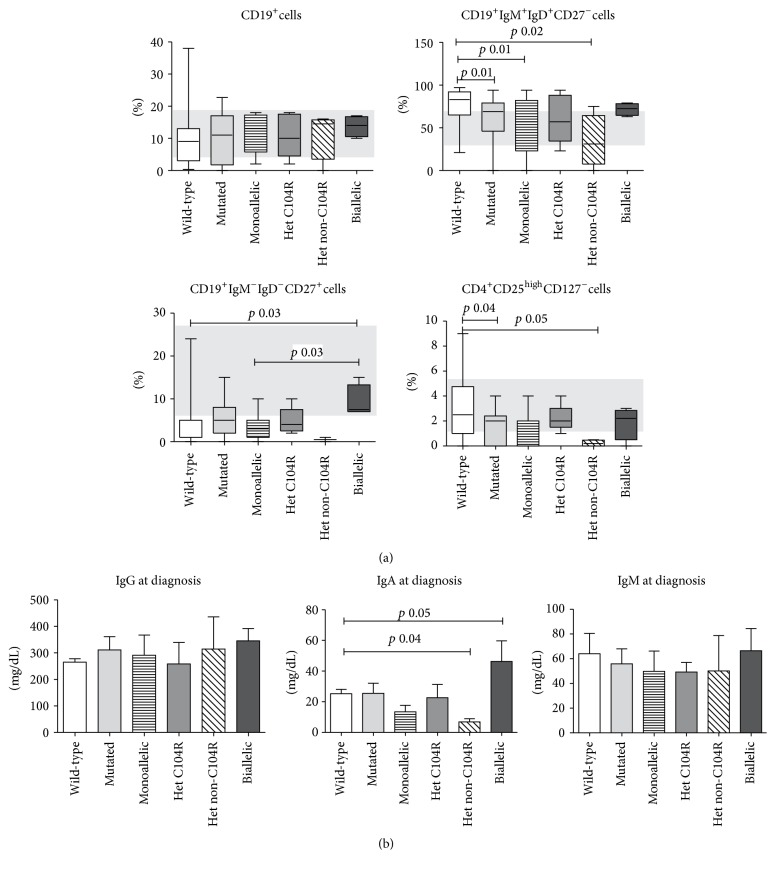
Immunological phenotype (a) and immunoglobulin levels (b) in TACI-deficient individuals and wild-type TACI patients. TACI-deficient individuals were grouped as carrying monoallelic TACI mutations (heterozygous C104R and heterozygous non-C104R) and biallelic TACI mutations. The grey area represents the referral values (http://www.ipidnet.org/).

Overall, TACI-mutated patients had a lower frequency of Treg cells than CVID wild-type subjects (1.7%  ± 1.3 versus 2.9%  ± 2.2, *p* = 0.04). In particular, the group carrying non-C104R heterozygous TACI mutations had the lowest Treg subset frequency (0.1%  ± 0.1, *p* = 0.05). No difference was observed between CVID with TACI wild-type sequence and CVID carrying biallelic mutations on the frequency of other T cells subsets. Immunophenotype data are summarized in Figure 3(a).

Serum immunoglobulin levels at diagnosis were similar in TACI-mutated and TACI wild-type CVID (Figure 3(b)). The group carrying non-C104R* TNFRSF13B* mutations had the lowest IgA levels compared to wild-type subjects (25.2 ± 31.3 versus 6.8 mg/dL ± 5.5, *p* = 0.04).

*(b) Biallelic TNFRSF13B Mutations*. Biallelic TACI-mutated CVID had higher frequency of switched memory B-cells compared to TACI wild-type CVID patients (9.25%  ± 3.8 versus 3.5 ± 5.0, *p* = 0.03) but similar frequency of naïve B-cells. Biallelic TACI-mutated CVID had also higher frequency of switched memory B-cells in comparison with patients with monoallelic TACI mutations (3.5%  ± 3.3, *p* = 0.03). No differences were found in the frequency of other B-cell subsets (data not shown). Moreover, patients carrying two mutated* TNFRSF13B* alleles had higher IgA levels than TACI wild-type CVID (25.2 mg/dL ± 31.3, 46.3 mg/dL ± 35.7, *p* = 0.05).

### 3.5. Antibody Response to Pneumococcal Polysaccharide Antigens in CVID

Due to the fact that all patients were under IgG replacement at the time of the study, we analyzed IgM and IgA antibodies to 23-valent polysaccharide antigens. At baseline, CVID patients carrying biallelic* TNFRSF13B* mutations had higher IgM anti-23-valent polysaccharide antigens compared to wild-type CVID patients (19.2 U/mL ± 7.0 versus 3.8 U/mL ± 7.1, *p* = 0.01) and compared to CVID carrying monoallelic* TNFRSF13B* mutations (3.9 U/mL ± 9.1, *p* = 0.05). The same was observed for specific IgA: CVID patients carrying biallelic mutations had higher prevaccination IgA anti-23-valent polysaccharide antigens compared to wild-type CVID (7 mU/mL ± 8 versus 0.3 mU/mL ± 0.8, *p* < 0.0001).

As expected, all CVID patients had a low vaccination response. Only biallelic-mutated patients had a significant increase in postvaccination IgM and IgA titers (24.0 U/mL ± 2.5, *p* = 0.05; 15 mU/mL ± 14, *p* = 0.05, resp.) while CVID carrying monoallelic-mutated or wild-type* TNFRSF13B* sequences had a mild but not significant increase of specific IgM (8.0 U/mL ± 12.1 and 5.0 ± 8.2, resp.) and of specific IgA (0.5 mU/mL ± 0.9 and 1.5 mU/mL ± 5, resp.).

### 3.6. Clinical Phenotypes and Serum Immunoglobulin Levels in TACI-Mutated IgAD

We found no differences in the spectrum of clinical manifestations between IgAD patients carrying TACI mutations and those with wild-type TACI. Clinical details of 9 TACI-mutated IgAD patients are reported in Table 5. Serum immunoglobulin levels at diagnosis were similar in TACI-mutated and wild-type TACI IgAD.

## 4. Discussion

TACI mutations have been identified in patients affected by a variety of clinical conditions including primary antibody deficiencies, sarcoidosis, and tonsillar hypertrophy [35]. In PADs, TACI has been analyzed in several cohorts from different geographical areas and mainly in CVID patients among whom the prevalence of mutations is 5–10%. Considering only mutations in exons 3 and 4, genetic alterations were found in about 10% of the CVID patients and in about 5% of the IgAD patients, significantly more than in the corresponding general population. We found a higher rate of TACI mutations ranging from 11.1% in CVID to 13.4% in symptomatic IgAD. The calculated relative risk was also higher ranging from 8.5 (CVID) to 10.3 (IgAD). In the two PAD groups, the majority of TACI mutations were monoallelic, with heterozygous C104R accounting for 2.3% (CVID) and 4.9% (IgAD). Surprisingly, in our series the frequency of monoallelic C104R mutation was not significantly different to that found in the cohort of Italian healthy donors while the frequency of autoimmune and lymphoproliferative signs overlapped that reported in the literature in CVID patients with monoallelic C104R mutations [12]. In summary, in Italian PADs we found pattern of TACI mutations different from other studies showing that ninety percent of all CVID associated* TNFRSF13B* mutations consist of either the C104R mutation, which alters ligand binding, or the A181E mutation that affects transmembrane function [30, 36].

The link to IgA deficiency is controversial. When first reported, TACI mutations were associated with CVID and IgA deficiency [10, 11] and a significant association of mutated* TNFRSF13B* gene and IgAD was later seen in other patients cohort [13]. On the other hand, Pan-Hammarström et al. showed that the* TNFRSF13B* variants have only minor roles, if any, in the development of selective IgAD [31]. IgAD may be an asymptomatic condition; therefore, establishing a prevalence of* TNFRSF13B* gene mutations in IgAD patients is challenging. The IgAD population described here included only symptomatic patients. In this case, a significant association of mutated* TNFRSF13B* gene and IgAD was strictly dependent on the choice of the healthy donors population. We have recruited healthy adult donors from Blood Donor Centers and we have chosen individuals >50 years old, in order to reduce the risk of the presence of an asymptomatic form of PAD. We found that near 4% of Italian CVID carried biallelic* TNFRSF13B* mutations. Moreover, we confirmed the previous data that TACI biallelic mutations are found only in CVID [12]. Thus, from the genetic point of view, the presence of biallelic mutations might correspond to a definitive diagnosis of CVID. However, from the clinical point of view, the analysis of clinical phenotypes confirmed the proposition that “two mutations are better than one” [16, 37]. In fact, patients with biallelic TACI mutations had a similar incidence of autoimmunity and lymphoproliferation signs compared to wild-type TACI patients, endorsing the hypothesis that if monoallelic TACI mutations reduce the elimination of autoreactive B-cells during the establishment of central B-cell tolerance, biallelic TACI mutations repress B-cell activation, preventing the development of autoimmunity. As shown by Salzer et al. [12], we confirmed that CVID patients carrying biallelic TACI mutations had higher relative frequency of switched memory B-cells. Moreover, we observed that biallelic TACI-mutated CVID patients had higher IgA serum levels at diagnosis and higher prevaccination and postvaccination IgM and IgA anti-pneumococcal polysaccharide antigens. Here, we analyzed the IgM- and IgA-mediated responses to TI-2 antigens. To the best of our knowledge, no data are available about TI-2 humoral response in humans carrying TACI mutations. Actually, in animal models the role of TACI in TI-2 antibody responses remains controversial. In murine knockout models, TACI is required for TI antibody responses to bacterial-associated polysaccharides [21–23] but is not crucial for TI antibody responses to whole bacteria [24]. Our data suggest that in biallelic-mutated CVID TI-2 antibody response is poor but less affected than in TACI monoallelic-mutated CVID. This was also confirmed by the observations that CVID carrying two TACI mutations did not show more severe and more recurrent infective manifestations than CVID having a TACI wild-type and monoallelic-mutated sequence.

Further studies are needed to unravel the additional genetic and environmental factors acting in concert with biallelic genetic alterations in* TNFRSF13B* to give rise to antibody deficiencies in humans.

Interestingly, the majority of patients with TACI-mutated PAD had monoallelic mutations non-C104R, detected in five per cent of CVID patients and about nine per cent of IgAD patients. CVID patients carrying monoallelic non-C104R mutations had a severe clinical phenotype with the highest prevalence within the CVID groups of autoimmunity, AHA, lymph nodes enlargement, and granulomatous diseases. These clinical features were associated with immunological abnormalities such as a low frequency of naïve B-cells, very low IgA serum levels, and a very low frequency of T regulatory cells. This last observation confirms already published data showing that defects in peripheral B-cell tolerance in monoallelic TACI-mutated CVID patients correlated with elevated plasma BAFF concentrations and decreased Treg frequencies [16]. These data further support the demonstration that defects in peripheral B-cell tolerance are correlated with altered T regulatory (Treg) cell frequency and function, a finding associated with clinical autoimmunity in CVID patients [38]. Thus, in comparison with other published data our cohort had a lower number of patients with monoallelic C104R mutation, a higher number of CVID patients carrying monoallelic mutations non-C104R, and a higher number of patients with compound heterozygous mutations.

The major challenge in diagnostic testing concerns the interpretation of the unknown variants, the so-called “Variants of Uncertain Significance” (VUS). As shown in Table 2, the majority of nonsynonymous variants of TACI are VUS. This stresses the importance of appropriate pretest counseling and informed consent by knowledgeable genetics professionals.

Despite consolidated evidence for a pathogenic role of specific* TNFRSF13B* mutations, such as C104R and A181E, in B-cell tolerance [16] and impaired antibody production due to haploinsufficiency during the later stages of B-cell development [20], further work is required to identify additional genetic hits and their potential clinical impact. This suggests caution in the introduction of TACI genetic analysis in the diagnostic workup of PAD.

In conclusion,* TNFRSF13B* genetic screening of hypogammaglobulinemic patients may allow the identification of novel mutational patterns, offering insight into the biological mechanisms underlying the pathogenesis of PADs. However, as with counseling for risk assessment, geneticists should be aware of the fact that the interpretation of genetic testing for TACI mutations is difficult and the potential impact on clinical management is still limited.

## Figures and Tables

**Table:** (a) CVID

Patient subset (*n*)	Genotype	*N* (%)	*p* ^*∗*^	Relative risk (95% CI)
All patients (189)	Any mutation	21 (11.1%)	<0.0001	8.5 (2.6–28.1)
Patients with biallelic mutations excluded (181)	Monoallelic mutations	13 (7.2%)	0.0034	5.5 (1.56–19.0)
Patients with biallelic and C104R mutations excluded (177)	Monoallelic mutations excluding C104R	9 (5.1%)	0.0061	11.6 (1.5–91.0)
Patients with monoallelic mutations excluded (176)	Biallelic mutations	8 (4.5%)	0.0012	21.9 (1.3–376.9)
Patients without mutations other than heterozygous C104R (172)	Heterozygous C104R	4 (2.3%)	0.4084	2.7 (0.5–14.4)

**Table:** (b) IgAD

Patient subset (*n*)	Genotype	*N* (%)	*p* ^*∗*^	Relative risk (95% CI)
All IgAD patients (67)	Any mutation	9 (13.4%)	0.0001	10.3 (2.9–37.0)
Patients without mutations other than heterozygous C104R (64)	Monoallelic mutations excluding C104R	6 (9.4%)	0.0005	21.4 (2.6–174.3)
Patients without mutations other than heterozygous C104R (61)	Heterozygous C104R	3 (4.9%)	0.0064	5.6 (1.0–33.0)

^*∗*^Two-tailed *p* values calculated by Fisher's exact test.

**Table 2:** Summary of the nonsynonymous variants identified in patients with CVID and IgAD.

Variants	dbSNP	MAF (ExAC)^*∗*^	ClinVar	PolyPhen score
D41G	rs763197017	T < 0.0001	VUS^*∗∗*^	0.995, probably damaging
R72H	rs55916807	T = 0.0017	VUS	0.003, benign
I87N	rs72553877	T < 0.0001	VUS	0.986, probably damaging
C104Y	rs72553879	A < 0.0001	VUS	1.000, probably damaging
C104R	rs34557412	C = 0.003	Pathogenic allele	1.000, probably damaging
P151L	rs200037919	A < 0.0001	VUS	0.728, possibly damaging
C172Y	rs751216929	A < 0.0001	VUS	0.985, probably damaging
A181E	rs72553883	A = 0.005	Pathogenic allele	0.890, possibly damaging
G190A	Not described	n.d.	VUS	0.989, probably damaging
R202H	rs104894649	A < 0.0001	VUS	0.474, possibly damaging

MAF: minor allele frequency.

^*∗*^MAF source: Exome Aggregation Consortium (ExAC).

^*∗∗*^VUS: Variants of Uncertain Significance.

**Table 3:** Clinical summary of 21 CVID individuals with mutations in *TNFRSF13B*.

*n*.	Age	Sex	IgG^*∗*^	IgA^*∗*^	IgM^*∗*^	Autoimmunity			Lymphoproliferation			Cancer	Mutation
						AHE	ITP	Other	LNE	Spl	Gran		
I.20	48	F	551	97	115	No	Yes		No	No	No		c.[260 T>A];[542 C>A] p.[I87N];[A181E]
I.22	87	F	75	20	28	No	No		Yes	Yes	Yes		c.[311 G>A];[=] p.[C104Y];[=]
I.23	29	M	486	53	59	No	Yes	IBD	Yes	Yes	Yes		c.[310 T>C];[=] p.[C104R];[=]
I.34	60	F	44	0	0	No	No		Yes	Yes	No		c.[122 A>G];[=] p.[D41G];[=]
I.38	76	F	Na	Na	Na	Yes	No		No	Yes	No	Pancreatic carcinoma	c.[706 G>T];[=] p.[E236X];[=]
I.40	42	M	269	15	73	No	No		No	No	No		c.[310 T>C];[=] p.[C104R];[=]
I.45	49	M	157	7	6	Yes	No		Yes	No	Yes		c.[579 C>A];[=] p.[C193X];[=]
I.59	25	M	Na	Na	Na	No	No		No	No	No		c.[310 T>C];[542 C>A] p.[C104R];[A181E]
I.74	69	M	208	0	33	No	No		No	No	No	NHL	c.[204_205insA];[215 G>A] p.[L69TfsX12];[R72H]
I.75	53	F	356	72	33	No	No		Yes	Yes	No	Ovaric Theratoma	c.[310 T>C];[310 T>C] p.[C104R];[C104R]
I.77	25	F	430	8	152	Yes	Yes		No	Yes	No		c.[260 T>A];[=] p.[I87N];[=]
I.78	19	M	983	18	169	No	Yes		No	No	No		c.[260 T>A];[=] p.[I87N];[=]
I.82	49	M	380	0	39	No	No		No	Yes	No		c.[310 T>C];[=] p.[C104R];[=]
I.86	23	F	280	5	4	No	No		Yes	Yes	Yes		c.[492 C>G];[=] p.[Y164X];[=]
I.102	42	F	220	5	16	Yes	No		Yes	Yes	Yes		c.[ 515 G>A];[=] p.[C172Y];[=]
I.104	82	F	380	54	24	No	No		Yes	Yes	No	Sarcomas	c.[260 T>A];[492 C>G] p.[I87N];[Y164X]
I.118	54	F	240	5	140	No	Yes	RA	Yes	Yes	Yes		c.[492 C>G];[542 C>A] p.[Y164X];[A181E]
I.126	47	F	250	64	90	No	No		No	No	No		c.[306 C>G];[431 C>G,579 C>A] p.[Y102X][S144X,C193X]
I.138	31	M	90	5	4	No	Yes	Vitiligo	No	Yes	No		c.[542 C>A];[=] p.[A181E];[=]
I.152	58	F	82	25	47	No	Yes		Yes	Yes	No		c.[310 T>C];[=] p.[C104R];[=]
I.162	46	F	434	32	30	No	No		No	Yes	No		c.[310 T>C];[579 C>A] p.[C104R];[ C193X]

M: male, F: female, AHA: autoimmune hemolytic anemia, ITP: idiopathic thrombocytopenic purpura, LNE: lymph nodes enlargement; Spl: splenomegaly, Gran: granulomas, CD: coeliac disease, NHL: non-Hodgkin lymphoma, RA: rheumatoid arthritis, and Na: not available.

^*∗*^At diagnosis.

**Table 4:** Frequencies of clinical complications in patients with wild-type and TACI-mutated CVID.

	Wild-type *n* = 168	TACI mutated *n* = 21	*p* value
Autoimmunity, *n* (%)	**39 (23)**	**10 (48)**	**0.03**
Autoimmune cytopenia	22 (13)	9 (43)	0.002
Other autoimmune manifestations	27 (16)	3 (14)	ns
Lymphoproliferation, *n* (%)	**101 (60)**	**15 (71)**	**ns**
Splenomegaly	96 (57)	14 (67)	ns
Lymphadenopathy	41 (25)	10 (48)	0.03
Granulomatous disease	19 (11)	6 (29%)	0.04
Malignant lymphoproliferation	15 (19)	1 (4%)	ns
Cancer, *n* (%)	**27 (20)**	**4 (17)**	**ns**
Bronchiectasis, *n* (%)	**72 (43)**	**10 (53)**	**ns**
Chronic diarrhea, *n* (%)	**42 (25)**	**5 (25)**	**ns**

**Table 5:** Clinical summary of 9 IgAD individuals with mutations in *TNFRSF13B*.

*n*.	Age	Sex	IgG mg/dL^*∗*^	IgA mg/dL^*∗*^	IgM mg/dL^*∗*^	Autoimmunity			Lymphoproliferation			Cancer	Mutation
						AHA	ITP	Other	LNE	Spl	Gran		
I.224	4	M	1077	6	55	No	No		No	No	No	No	A181E hetero
I.192	7	M	794	29	110	No	No	CD	No	No	No	No	C104R hetero
I.193	19	M	1723	5	131	No	No		No	No	No	No	C104R hetero
I.194	24	F	1345	33	194	No	No	CD	No	No	No	No	C104R hetero
I.254	6	F	1024	4	73	No	No		No	No	No	No	C193X hetero
I.216	14	F	1370	3	140	No	No		No	No	No	No	C66X hetero
I.205	8	M	1601	4	102	No	No		No	No	No	No	G190A hetero
I.212	6	M	1139	5	46	No	No		No	No	No	No	P151L hetero
I.217	5	F	972	21	139	No	No		No	No	No	No	R202H hetero

M: male; F: female; AHA: autoimmune hemolytic anemia; ITP: idiopathic thrombocytopenic purpura; LNE: lymph nodes enlargement; CD: coeliac disease.

^*∗*^At diagnosis.
